# Deciphering Adverse Drug Reactions: *In Vitro* Priming and Characterization of Vancomycin-Specific T Cells From Healthy Donors Expressing HLA-A*32:01

**DOI:** 10.1093/toxsci/kfab084

**Published:** 2021-06-27

**Authors:** Monday O Ogese, Adam Lister, Joshua Gardner, Xiaoli Meng, Ana Alfirevic, Munir Pirmohamed, B Kevin Park, Dean J Naisbitt

**Affiliations:** Department of Molecular & Clinical Pharmacology, MRC Centre for Drug Safety Science, University of Liverpool, Liverpool L69 3GE, UK

**Keywords:** drug hypersensitivity, vancomycin, T cells, HLA

## Abstract

Drug rash with eosinophilia with systemic symptoms (DRESS) is a serious adverse event associated with use of the glycopeptide antibiotic vancomycin. Vancomycin-induced drug rash with eosinophilia with systemic symptoms is associated with the expression of human leukocyte antigen (HLA)-A*32:01, suggesting that the drug interacts with this HLA to activate CD8+ T cells. The purpose of this study was to utilize peripheral blood mononuclear cell from healthy donors to: (1) investigate whether expression of HLA-A*32:01 is critical for the priming naïve of T cells with vancomycin and (2) generate T-cell clones (TCC) to determine whether vancomycin exclusively activates CD8+ T cells and to define cellular phenotype, pathways of drug presentation and cross-reactivity. Dendritic cells were cultured with naïve T cells and vancomycin for 2 weeks. On day 14, cells were restimulated with vancomycin and T-cell proliferation was assessed by [^3^H]-thymidine incorporation. Vancomycin-specific TCC were generated by serial dilution and repetitive mitogen stimulation. Naïve T cells from HLA-A*02:01 positive and negative donors were activated with vancomycin; however the strength of the induced response was significantly stronger in donors expressing HLA-A*32:01. Vancomycin-responsive CD4+ and CD8+ TCC from HLA-A*32:01+ donors expressed high levels of CXCR3 and CCR4, and secreted IFN‐γ, IL-13, and cytolytic molecules. Activation of CD8+ TCC was HLA class I-restricted and dependent on a direct vancomycin HLA binding interaction with no requirement for processing. Several TCC displayed cross-reactivity with teicoplanin and daptomycin. To conclude, this study provides evidence that vancomycin primes naïve T cells from healthy donors expressing HLA-A*32:01 through a direct pharmacological binding interaction. Cross-reactivity of CD8+ TCC with teicoplanin provides an explanation for the teicoplanin reactions observed in vancomycin hypersensitive patients.

Vancomycin is a complex glycopeptide antibiotic obtained from *Streptomyces orientalis* which inhibits Gram positive bacterial cell wall synthesis. Over the years, other potent semisynthetic glycopeptides have been introduced into the clinic for the treatment of drug-resistant bacterial infections. These include dalbavancin, oritavancin, daptomycin, teicoplanin, ramoplanin, decaplanin, corbomycin, bleomycin, and telavancin (Blaskovich *et al.*, 2018). In addition to their primary clinical application, glycopeptide antibiotics are recommended for use in β-lactam hypersensitive patients (Okano *et al.*, 2017). Vancomycin exposure is associated with the development of several forms of delayed-type adverse event. Of particular concern is drug reaction with eosinophilia and systemic symptom (DRESS), which has a delayed onset (2–6 weeks) and is characterized by fever, eosinophilia, renal impairment, severe skin reactions, lymphadenopathy, and/or liver dysfunction (Konvinse *et al.*, 2019; Littlehales *et al.*, 2018; Minhas *et al.*, 2016). CD4+ and CD8+ T cells activated by a range of drugs have been detected in blood of patients with DRESS (Mauri-Hellweg *et al.*, 1995; Zhao *et al.*, 2019). Furthermore, granzyme B secreting cytotoxic T cells have been detected next to apoptotic hepatocytes in inflamed liver, in the acute phase of a sulfasalazine-induced DRESS after additional vancomycin treatment (Mennicke *et al.*, 2009).

Genome-wide association studies (GWASs) have linked the expression of human leukocyte antigen ( HLA)-A*32:01 allele to vancomycin-induced DRESS (Konvinse *et al.*, 2019) and a rapid real-time polymerase chain reaction method has been developed to prospectively identify HLA-A*32:01 expression and the risk of developing vancomycin-induced DRESS (Rwandamuriye *et al.*, 2019). Approximately 7% of individuals of European ancestry express HLA-A*32:01 allele and about 20% of these individuals will experience DRESS as a result of vancomycin treatment. These data suggests that vancomycin might preferentially interact with the protein encoded by HLA-A*32:01 or peptides displayed by the HLA-A*32:01 protein to activate vancomycin-specific T cells. Recently, Nakkam *et al.* (2021) have shown that peripheral blood mononuclear cells (PBMC) from patients with vancomycin-induced DRESS secrete IFN-γ when stimulated *in vitro* with the drug; however, the nature of the induced response as well as pathways of vancomycin-specific T-cell activation remain ill-defined.

*In silico* molecular docking studies show a potential binding interaction between a peptide occupying the HLA-A*32:01 antigen binding cleft and vancomycin. However, a greater binding affinity was predicted when the drug was docked to the HLA protein in the absence of peptide (Konvinse *et al.*, 2019). These data suggest that vancomycin may activate T cells via a pharmacological interactions (p-I) pathway (Pichler, 2002) with the T-cell receptor receiving signals from the HLA molecule, the bound peptide and vancomycin, but intriguingly also indicate that vancomycin with a heptapeptide core structure might actually act as a peptidomimetic displacing the peptide from the HLA-A*32:01 binding cleft.

To date, about 25 risk HLA alleles have been associated with adverse drug reactions targeting the skin, liver, blood, and other organ systems (Gibson *et al.*, 2018). This list will increase as new drugs are introduced into clinic or already licensed drugs are studied more extensively. The preprescription HLA-B*57:01 screening of patients before initiating abacavir therapy has reduced the incidence of hypersensitivity reactions and this is viewed as an important milestone for personalized medicine (Mallal *et al.*, 2008). The association between the expression of HLA-B*57:01 allele and abacavir hypersensitivity syndrome was first reported in 2002 by 2 independent studies (Hetherington *et al.*, 2002; Mallal *et al.*, 2002). However, the exact molecular mechanism was defined 10 years later (Illing *et al.*, 2012; Norcross *et al.*, 2012; Ostrov *et al.*, 2012), when abacavir was found to interact via noncovalent bonds with the F-pocket of the peptide-binding groove of HLA-B*57:01 protein leading to alteration of self-peptides displayed on the surface of antigen presenting cells for presentation to T cells. The success of abacavir HLA-B*57:01 screening derives from the 100% negative predictive value (ie, reactions only occur in individuals with the allele), coupled with a positive predictive value of 55% (ie, donors expressing HLA-B*57:01 that develop hypersensitivity when exposed to abacavir) for the association. This shows that expression of HLA-B*57:01 is essential for hypersensitivity reactions to develop, but alone is not sufficient. Other HLA-linked adverse reactions have much lower positive predictive values making predictive screening and development of mechanistic approaches to study pathways of T-cell activation more difficult.

To overcome this problem, our study of vancomycin-specific T-cell responses utilized a recently developed T-cell multi-well assay to investigate the priming of naïve T cells isolated from our biobank of HLA-typed healthy donor PBMC (Alfirevic *et al.*, 2012; Ogese *et al.*, 2020). Vancomycin-responsive T-cell clones (TCC) were then generated from 3 HLA-A*32:01 positive individuals and profiled for their phenotype (CD4/CD8 marker and chemokine receptor expression), function, pathways of vancomycin-specific T-cell activation and cross-reactivity with structurally related drugs.

## MATERIALS AND METHODS

###  

####  

##### Human subjects

Blood donated by 18 HLA-typed healthy donors were utilized for this study. Table 1 shows the HLA type of the donors. Antigen presenting cells from all donors were used in HLA mismatching experiments. PBMC from 3 HLA-A*32:01 positive (donors 4, 5 and 7) and 3 HLA-A*32:01 negative (donors 1, 2, and 3) were used in vancomycin naïve T-cell priming experiments. Donors 4, 5, and 7 were used to generate vancomycin-responsive TCC. Each individual donated 100 ml of blood. All the blood donors had given informed written consent to partake in this study approved by the Liverpool local research ethics committee. Blood was drawn into lithium heparin coated tubes.

**Table 1.** HLA Genotype of Healthy Donors for HLA Mismatch Assay

	HLA Class I						HLA Class II					
Subject ID	HLA-A		HLA-B		HLA-C		HLA-DRB1		HLA-DQB1		HLA-DQA1	
Donor 1	A*02:01	A*03:01	B*44:03	B*44:02	C*05:27	C*16:01	DRB1*07:01	DRB1*15:01	DQB1*02:01	DQB1*06:02	DQA1*01:02	DQA1*01:01
Donor 2	A*02:30	A*31:01	B*38:01	B*39:01	C*07:01	C*12:03	DRB1*01:01	DRB1*13:01	DQB1*05:01	DQB1*06:03	DQA1*01:03	DQA1*01:02
Donor 3	A*24:02	A*25:01	B*15:01	B*57:01	C*03:03	C*06:02	DRB1*04:02	DRB1*07:01	DQB1*03:02	DQB1*03:03	DQA1*02:01	DQA1*03:01
Donor 4*a*	A*01:01	A*32:01	B*08:01	B*08:01	C*07:01	C*08:02	DRB1*03:01	DRB1*11:01	DQB1*02:01	DQB1*03:01	DQA1*05:01	DQA1*05:01
Donor 5	A*01:01	A*32:01	B*44:02	B*57:01	C*05:01	C*06:02	DRB1*07:01	DRB1*12:01	DQB1*03:01	DQB1*03:02	DQA1*02:01	DQA1*05:01
Donor 6	A*02:01	A*32:01	B*14:01	B*44:02	C*05:01	C*08:02	DRB1*07:01	DRB1*13:01	DQB1*02:01	DQB1*06:03	DQA1*01:03	DQA1*02:01
Donor 7*b*	A*03:01	A*32:01	B*07:02	B*51:01	C*07:02	C*15:02	DRB1*01:01	DRB1*11:04	DQB1*03:01	DQB1*05:01	DQA1*01:01	DQA1*05:01
Donor 8	A*01:01	A*32:01	B*08:01	B*27:05	C*01:02	C*07:01	DRB1*01:01	DRB1*03:01	DQB1*02:01	DQB1*05:01	DQA1*01:01	DQA1*05:01
Donor 9	A*03:01	A*32:01	B*07:02	B*44:02	C*03:03	C*07:02	DRB1*13:01	DRB1*15:01	DQB1*06:02	DQB1*06:03	DQA1*01:03	DQA1*01:02
Donor 10	A*03:01	A*32:01	B*35:01	B*44:03	C*04:01	C*16:01	DRB1*01:01	DRB1*07:01	DQB1*02:01	DQB1*05:01	DQA1*01:01	DQA1*02:01
Donor 11	A*26:01	A*32:01	B*44:02	B*51:01	C*05:01	C*15:02	DRB1*11:01	DRB1*12:01	DQB1*03:01	DQB1*03:01	DQA1*05:01	DQA1*05:01
Donor 12	A*02:01	A*32:01	B*15:01	B*44:02	C*03:03	C*05:01	DRB1*04:01	DRB1*15:01	DQB1*03:02	DQB1*03:01	DQA1*03:01	DQA1*05:01
Donor 13	A*03:01	A*32:01	B*14:02	B*27:05	C*01:02	C*08:02	DRB1*01:01	DRB1*15:01	DQB1*05:01	DQB1*06:02	DQA1*01:02	DQA1*01:01
Donor 14	A*02:01	A*24:02	B*07:02	B*15:25	C*07:26	C*07:02	DRB1*15:01	DRB1*15:01	DQB1*06:01	DQB1*06:02	—	—
Donor15	A*11:01	A*31:01	B*40:02	B*40:01	C*03:03	C*03:04	DRB1*13:01	DRB1*14:01	DQB1*05:03	DQB1*06:03	DQA1*01:03	DQA1*01:01
Donor 16	A*01:01	A*02:01	B*45:01	B*51:01	C*01:02	C*06:02	DRB1*01:01	DRB1*07:01	DQB1*02:01	DQB1*05:01	DQA1*01:01	DQA1*02:01
Donor 17	A*11:01	A*30:01	B*13:02	B*35:01	C*04:01	C*06:02	DRB1*15:01	DRB1*16:01	DQB1*05:02	DQB1*06:03	DQA1*01:02	DQA1*01:02
Donor 18	A*01:02	A*26:01	B*40:01	B*49:01	C*03:04	C*07:01	DRB1*04:04	DRB1*07:01	DQB1*02:01	DQB1*03:02	DQA1*02:01	DQA1*03:01

a PBMC from healthy donors used to generate vancomycin TCC (donors 4, 5, and 7). PBMC from all other donors were used to generate HLA mis-matched antigen presenting cells.

b Autologous APC.

##### Isolation of PBMC

Briefly, blood was layered on top of lymphoprep (Axis-Shield PoC AA, Oslo, Norway) in a 1:1 volume ratio and centrifuged at 2000 rpm, for 25 min without brake. The PBMC buffy coat was collected using a Pasteur pipette and washed twice with HBSS buffer solution. Isolated cells were suspended in T-cell culture media composed of RPMI 1640 supplemented by penicillin (100 mg/ml), streptomycin (100 U/ml), transferrin (25 mg/ml), human AB serum (10%), HEPES buffer (25 mM), and L-glutamine (2 mM). All cell culture media supplements including RPMI and lipopolysaccharide (LPS) (O111: B4 from *Escherichia coli*) were purchased from Sigma-Aldrich, UK.

##### Priming of naïve T cells from HLA-A*32:01 negative and positive individuals

A T-cell multiple well assay was used to investigate the priming of naïve T cells isolated from either HLA-A*32:01 negative or HLA-A*32:01 positive donors (Ogese *et al.*, 2020). CD14+ monocytes were positively selected from total PBMC using CD14 antibody-conjugated microbeads. CD25+ cells were depleted from the non-CD14-fraction. Finally, CD45RA+ naïve T cells were isolated by negative selection. Naïve T cells with a purity of >97% were stored at −150°C freezer while CD14+ monocytes were used to generate dendritic cells. Antibody-conjugated magnetic beads were purchased from (Miltenyi Biotec, Gadbach, Germany). Dendritic cells were generated by culturing monocytes with a cocktail of GM-CSF (800 U/ml) and IL-4 (800 U/ml) in culture media for 6 days. Both cytokines were purchased from Peprotech, New Jersey. A maturation cocktail of TNF-α (25 ng/ml) and LPS (1 µg/ml, from *E.* *coli* serotype O111: B4, Sigma-Aldrich) was added directly to the cultures for 16 h. The matured dendritic cells were harvested and washed before establishing the T-cell priming culture. Briefly, dendritic cells (8 × 10^3^) were cultured with naïve T cells (1 × 10^5^) and vancomycin (0.5 mM) in a 96-well tissue culture plate for 14 days at 37°C, 5% CO_2_. After 2 weeks, medium was removed by gentle pipetting. Half of the cultures were then stimulated with vancomycin for 48 h. The other wells served as a medium control. [^3^H]-thymidine (0.5 µCi/well) was added for the final 16 h to assess vancomycin-induced T-cell proliferation. Plates were harvested using TomTec Harvester 96 (Receptor Technologies) onto filter mats, sealed with scintillation wax and T-cell activation determined using a MicroBeta TriLux 1450 LSC β-counter (PerkinElmer). The degree of naïve T-cell priming is displayed as (1) cpm counts in individual wells and (2) bar chats depicting the percentage of wells displaying the following responses: negative (stimulation index [SI = cpm of vancomycin-treated wells/average cpm of media-treated wells] <1.5), weak (SI = 1.5–1.99), good (SI = 2–3.99), strong (SI = 4–10), and extreme (SI = >10) response. Student’s *t* test was performed to determine statistical significance of T-cell proliferation when comparing medium- and vancomycin-treated cultures (*p ≤ .05; **p ≤ .005; ***p < .001).

##### Long-term stimulation of PBMC cultures from HLA-A*32:01 negative and positive individuals

To further explore T-cell responses to vancomycin in HLA-A*32:01 negative and positive donors, cryopreserved PBMC isolated from 20 HLA-typed healthy individuals (10 HLA-A*32:01 positive and 10 HLA-A*32:01 negative donors) were cultured in a 96-well plate at 2 × 10^5^ PBMC/well in medium containing vancomycin (0.5 mM) and IL-2 (50 U/ml, Peprotech). On day 3, cultures were supplemented with fresh medium containing IL-2. Restimulation of PBMC cultures was performed on day 7 and weekly thereafter in which wells were exposed to a cocktail containing irradiated autologous PBMC (5 × 10^3^/well), vancomycin (0.5 mM) and fresh IL-2. After 3 weeks, restimulated PBMC cultures were washed 5 times in cell culture medium to remove free soluble drug and then challenged with irradiated autologous PBMC (5 × 10^3^/well) in addition to either vancomycin (0.5 mM; up to 36 wells per condition, depending on cells available), medium or piperacillin (irrelevant drug antigen; 2 mM) for 48 h. [^3^H]-thymidine (0.5 µCi/well) was added for the final 16 h and plates were harvested to measure proliferation. Results are expressed as cpm in individual culture wells.

##### Generation of EBV-transformed B cells and vancomycin-specific T-cell lines and TCC

Epstein-Barr virus (EBV)‐transformed B‐cell lines were used as antigen presenting cells. Briefly, PBMC were cultured with supernatant from the EBV‐producing cell line B9‐58 overnight. The cells were then washed and maintained in culture medium consisting of RPMI supplemented with bovine serum albumin (10%) penicillin (100 mg/ml), streptomycin (100 U/ml), transferrin (25 mg/ml), human AB serum (10%), HEPES buffer (25 mM), and L-glutamine (2 mM). Cyclosporin A was included in the medium for the first 3 weeks to culture to prevent T-cell outgrowth.

To establish T-cell lines, PBMC from HLA-A*32:01+ donors were cultured with vancomycin (0.5 mM) in T-cell culture medium supplemented with IL-2 (50 U/ml) (Peprotech) for 14 days. Vancomycin-specific TCC were generated from the T-cell lines by serial dilution and repetitive mitogen stimulation (Schnyder *et al.*, 2000). Briefly, T cells (0.3–3 cells/well) were cultured with irradiated allogenic PBMC (5 × 10^4^/well) and phytohemagglutinin (PHA-P, 1 µg/ml; purchased from Sigma-Aldrich) in IL-2 containing medium. The medium was supplemented with fresh IL-2 on days 5 and 8. Growing cultures were expanded with a second round of mitogen stimulation and tested for vancomycin-specific T-cell proliferation through culture with irradiated autologous EBV-transformed B cells (5 × 10^4^/well) and vancomycin (0.5 mM) in duplicate wells for 48 h. Wells containing medium served as a negative control. Proliferation was measured by the addition of [^3^H]-thymidine followed by scintillation counting. TCC with SI ≥ 2 were considered vancomycin-responsive and expanded for dose-response assessment and functional assays. Human antibody-conjugated CD8 beads (130-045-201; purchased from Miltenyi Biotec) were used to enrich the CD8+ T-cell population prior to cloning in certain experiments.

##### Phenotypic analysis of vancomycin-responsive TCC

CD4/CD8 phenotype of TCC was determined using CD4-APC (clone RPA T4) and CD8-PE (clone HIT8a) fluorescence antibodies (BD Biosciences, Oxford, UK) by flow cytometry. Furthermore, TCC were profiled for the expression of chemokine receptors including CXCR3 (clone 49801), CCR5 (clone CTC5), CCR1 (clone 53504), CCR4 (clone 1G1), CCR9 (clone 248621), CCR8 (clone 191704), CCR6 (Clone 53103), CLA (clone HECA-452), CXCR6 (clone 56811), E-cadherin (clone 180224), CXCR1 (clone 42705), CCR2 (clone 48607), and CD69 (Clone 298614) using fluorescent antibodies purchased from R&D Systems (Minneapolis). Cells (10 000) were acquired using a FACSCanto II (BD Biosciences) and data analyzed using Cyflogic software.

##### [^3^H]-thymidine assay for assessment of proliferation of vancomycin-responsive TCC

Dose-dependent vancomycin-specific activation of TCC was performed by incubating TCC (5 × 10^4^/well) with irradiated autologous EBV-transformed B cells (1 × 10^4^/well) and graded concentrations of vancomycin (0.01–1 mM) for 48 h in triplicate wells at 37°C, 5% CO_2_. Vancomycin concentrations up to 1 mM did not inhibit the proliferation of PHA-stimulated PBMC. Proliferation was measured by the addition of [^3^H]-thymidine (0.5 µCi/well) followed by scintillation counting. Vancomycin-responsive TCC and irradiated autologous EBV-transformed B cells were also cultured with teicoplanin, dalbavancin, telavancin, or daptomycin (all 0.01–1 mM) for assessment of cross-reactivity. TCC and irradiated autologous EBV-transformed B cells in the absence of drug were used as a negative control.

##### ELIspot assay for assessment of cytokine and cytolytic molecule release from vancomycin-responsive TCC

Vancomycin-induced cytokine secretion from TCC was assessed using ELIspot assay. ELIspot plates were coated with IFN-γ, IL-5, IL-10, IL-13, IL-17, IL-22, perforin, granzyme B, and Fas Ligand capture antibodies overnight. Cytokine kits were purchased from Mabtech (Nacka Strand, Sweden). Plates were washed, blocked with T-cell culture media and TCC were incubated with autologous EBV-transformed B cells in the presence and absence (as negative control) of vancomycin for 48 h at 37°C, 5% CO_2_. The ELIspot plates were developed according to the manufacturer’s instructions and spots were counted using an AID ELIspot reader.

##### Assessment of pathways of vancomycin-specific T-cell activation

To assess pathways of TCC activation, the autologous EBV-transformed B cells were first omitted from the T-cell proliferation and ELIspot assays. Secondly, autologous EBV-transformed B cells were pulsed with vancomycin (0.5 mM) for 16 h prior to extensive washes to remove the unbound drug. The EBV-transformed B cells were then irradiated and co-cultured (1 × 10^4^) with vancomycin-specific TCC (5 × 10^4^) in the absence of soluble drug for 48 h, 37°C and 5% CO_2_. T-cell activation was measured by ^3^H-thymidine incorporation. TCC incubated with unpulsed autologous EBV-transformed B cells in the presence or absence of the soluble vancomycin were used as positive and negative controls, respectively. Third, glutaraldehyde-fixed EBV-transformed B cells were irradiated and cultured with TCC and vancomycin (0.5 mM) for 48 h and T-cell proliferation was determined as described earlier. Briefly, EBV-transformed B cells (2 × 10^6^ cells/ml) were washed in HBSS buffer to exclude foetal bovine serum (FBS) and resuspended in HBSS buffer (1 ml). Glutaraldehyde (25%, 1 µl) purchased from Sigma-Aldrich was added and the cells were gently mixed for 30 s. Glycine (1 ml of 1 M) was quickly added and the cell suspension was mixed for a further 45 s. Cells were then washed 3 times to remove glutaraldehyde and suspended in T-cell culture medium. Fixation blocks antigen processing, but not presentation of drugs displayed directly by HLA molecules expressed on the surface of antigen presenting cells (Zanni *et al.*, 1998).

To investigate whether activation of the TCC with vancomycin was HLA class I-restricted TCC were incubated with irradiated autologous EBV-transformed B cells and vancomycin in the presence and absence of antihuman HLA class I (DX17) and II (Tu39) blocking antibodies or isotype control class I (MOPC-21) and II (MOPC-21) antibodies purchased from BD Pharmingen (San Jose, USA). T-cell proliferative responses were measured using [^3^H] thymidine incorporation. Finally, vancomycin-responsive TCC were cultured with drug and either autologous (HLA-A*32:01+) or up to 14 heterologous EBV-transformed B cells expressing similar or dissimilar HLA-A* alleles (HLA type of the blood donors is shown in Table 1). TCC cultured with relevant EBV-transformed B cells in the absence of drug was used as a negative control. T-cell activation was measured after 48 h via [^3^H]-thymidine incorporation.

##### Statistics

Student’s *t* test was performed to determine statistical significance of T-cell proliferation (*p ≤ .05; **p ≤ .005; ***p < .001).

## RESULTS

###  

#### Naïve T-Cell Priming From HLA-A*32:01 Negative and Positive Were Primed to Vancomycin

**Figure 1.**
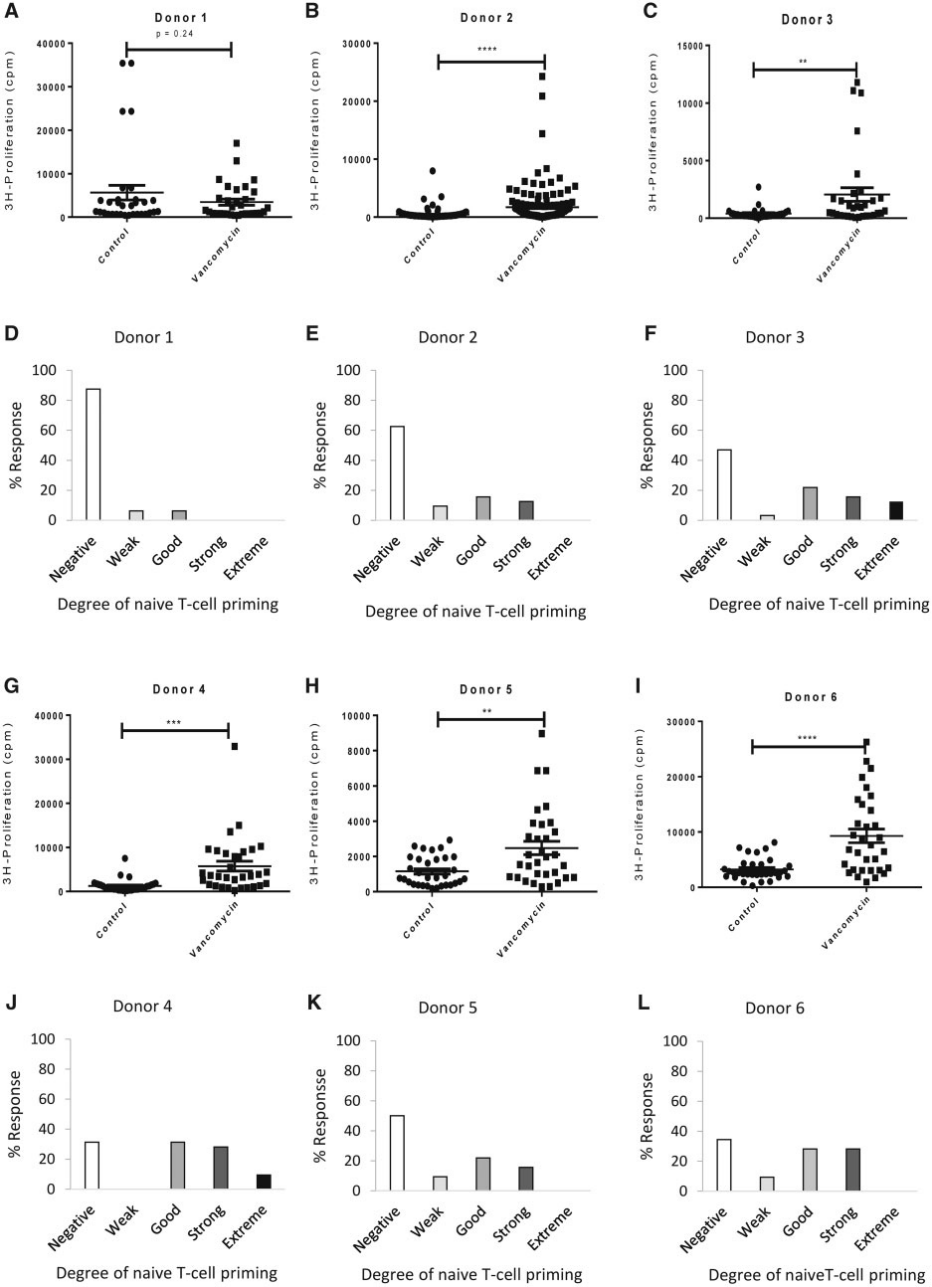
Priming of naïve T cells from HLA-A*32:01 negative and positive vancomycin naïve healthy donors. A–C, Naïve T cells (1 × 10^5^) from 3 HLA-A*32:01 negative healthy donors were incubated with autologous dendritic cells (8 × 10^3^) with vancomycin (0.5 m M) in a U-bottomed 96-well plate for 14 days. Plates were then washed extensively with cell culture media to remove free drug. Cells were gently resuspended in media, re-challenged with vancomycin (0.5 mM) or media (negative control) and incubated for 48 h. ^3^H-thymidine (0.5µCi/well) was added during the last 16 h before plates were harvested. T-cell activation was determined using a beta-counter (counts per minute, cpm). **D–F,** Stimulation index (SI) was calculated as cpm drug treated well/average cpm media treated wells. The degree of naïve T-cell priming is displayed as bar chats: Negative response (SI < 1.5), weak response (SI = 1.5–1.99), good response (SI = 2–3.99), strong response (4 SI = 4–10), and extreme response (SI > 10). Student’s *t* test was performed to determine statistical significance of T-cell proliferation (*p ≤ .05; **p ≤ .005; ***p < .001). G–I, Naïve T cells and dendritic cells from 3 HLA-A*32:01 positive healthy donors were cultured and assayed for T-cell priming using the approach described in (A–C). J–L, Analysis of the degree of naïve T-cell primed to vancomycin displayed as bar chats as in (D–F).

Vancomycin-specific activation of naïve T cells was observed in donors expressing the risk allele HLA-A*32:01 and in donors expressing other HLA-A alleles (Figure 1). Significant vancomycin-specific T-cell proliferative responses were detected from 2/3 donors that did not express HLA-A*32:01 (Figs. 1A–F), while responses were detected in all 3 donors expressing HLA-A*32:01 (Figs. 1G–L). Approximately 60.0% of naïve T cells from donors expressing the risk HLA-A*32:01 were primed to vancomycin compared with 34.6% priming in donors negative for the risk allele.

#### Activation of Long-Term PBMC Cultures From HLA-A*32:01 Negative and Positive Donors With Vancomycin

**Figure 2.**
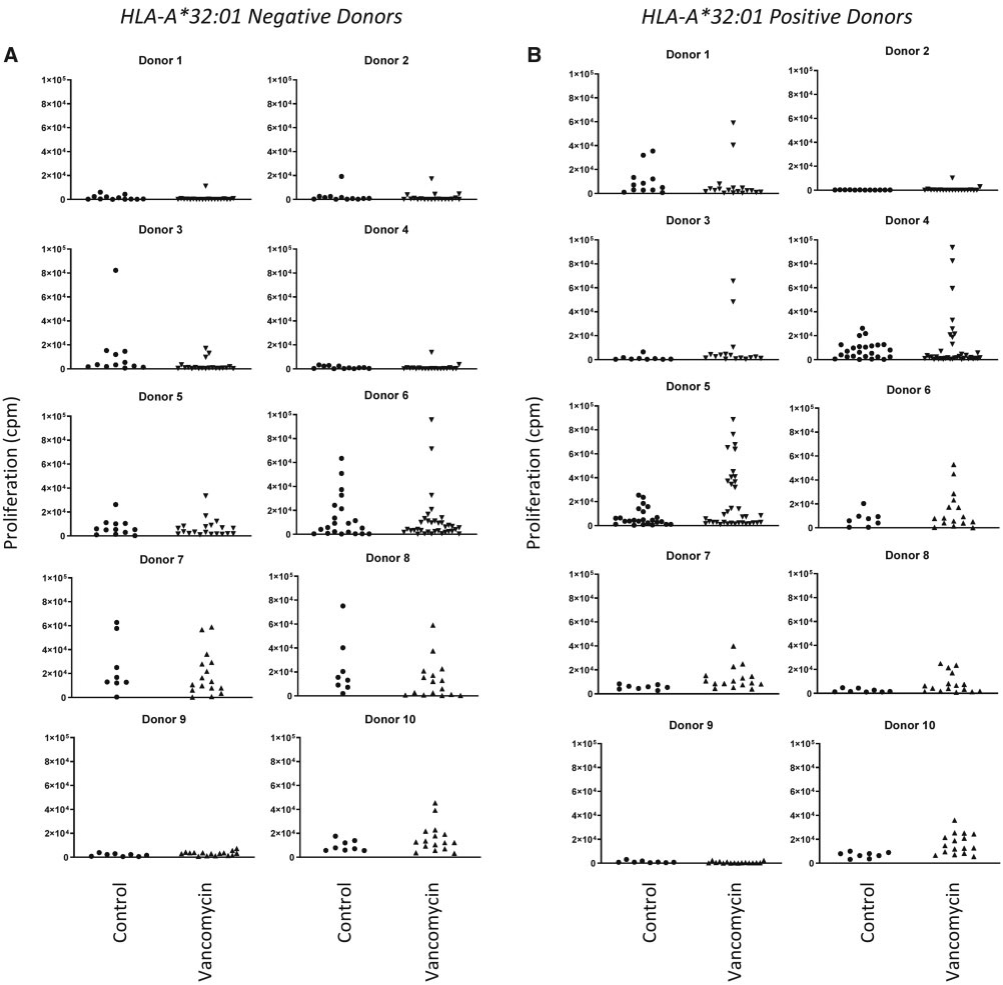
Vancomycin-specific activation of peripheral blood mononuclear cells (PBMC) from HLA-A*32:01 negative and positive healthy donors. PBMC (2 × 10^5^) from 10 HLA-A*32:01 negative healthy donors were cultured with vancomycin (0.5 mM) and IL-2 (50 U/ml) for 7 days. Restimulation of PBMC cultures was performed weekly using a cocktail containing irradiated autologous PBMC (5 × 10^3^/well), vancomycin (0.5 mM) and fresh IL-2. PBMC were then washed and rechallanged with irradiated autologous PBMC (5 × 10^3^/well) in addition to either vancomycin (0.5 mM) or medium for 48 h. [^3^H]-thymidine (0.5 µCi/well) was added for the final 16 h and plates were harvested to measure proliferation. B, PBMC from 10 HLA-A*32:01 positive healthy donors were cultured and assayed for T-cell responses using the approach described in (A). Results are expressed as cpm drug-treated and medium-treated wells.

PBMC from 10 HLA-A*32:01 positive and negative donors were cultured with vancomycin and repetitively restimulated with a cocktail containing autologous irradiated PBMC, vancomycin and IL-2 over a period of 4 weeks to enrich to number of vancomycin-responsive T cells. PBMC cultures were washed and individual wells treated with either medium or vancomycin before assessment of proliferation. Wells containing vancomycin-responsive T cells were detected in 7/10 HLA-A*32:01 positive donors (donors 3, 4, 5, 6, 7, 8, and 10) and 1/10 HLA-A*32:01 negative donor (donor 10, Figure 2; donor number in this figure does not relate to the HLA genotypes described in Table 1). The β-lactam antibiotic piperacillin was added to separate wells during the restimulation step as an irrelevant drug antigen. PBMC were not stimulated to proliferate with piperacillin (results not shown).

#### Characterization of Vancomycin-Specific CD4+ TCC Generated From HLA-A*32:01 Positive Drug Healthy Donors

**Figure 3.**
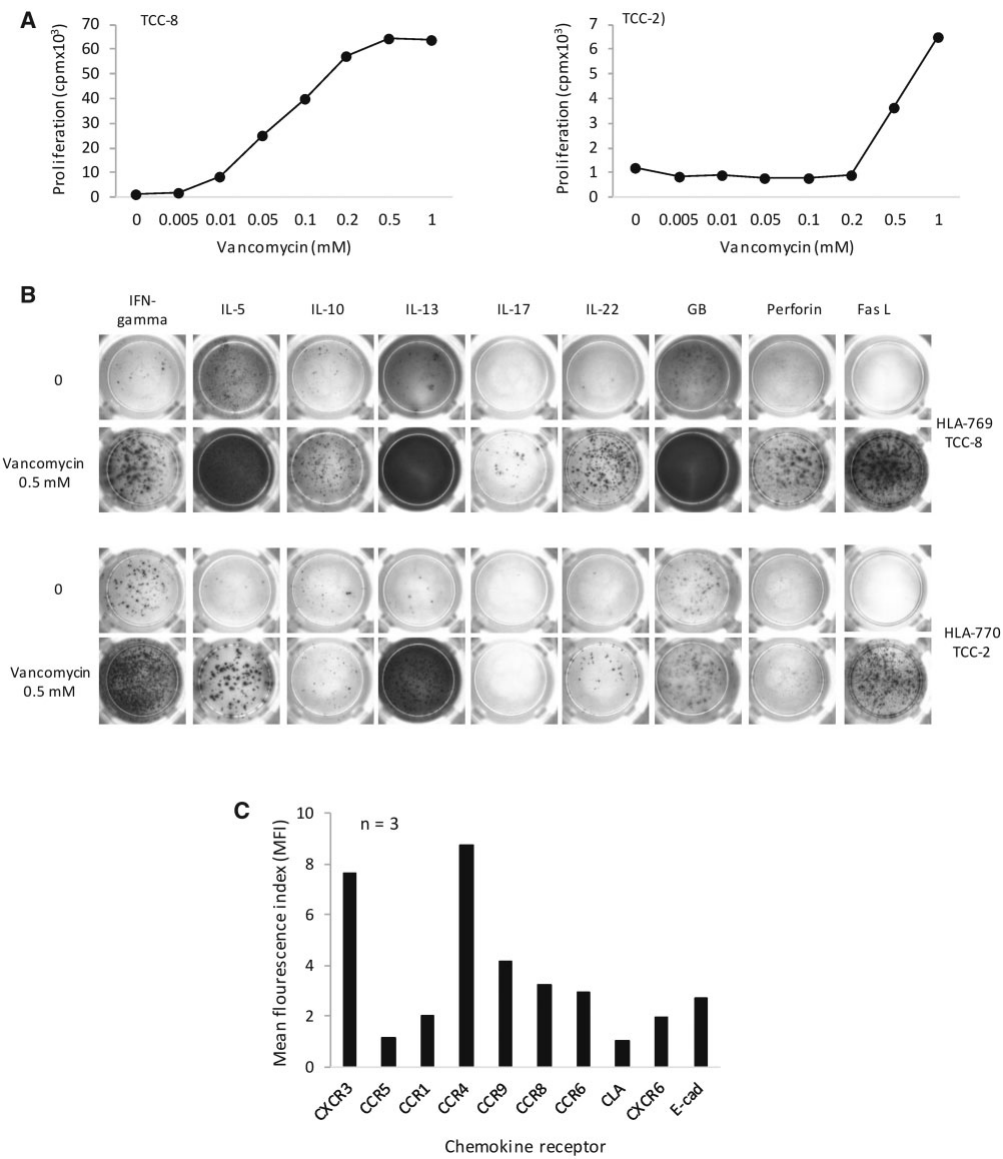
Vancomycin stimulates CD4+ T-cell clones (TCC) to proliferate and secrete Th1, Th2, and cytolytic molecules. A, Concentration-dependent proliferation of T cells. TCC (0.5 × 10^5^) cultured with autologous antigen presenting cells (0.1 × 10^5^) and vancomycin in triplicate cultures for 48 h and T-cell activation was measured by ^3^H-thymidine incorporation. B, ELISpot detection of IFN‐γ, IL-5, IL-10, IL‐13, IL‐17, IL‐22, perforin, granzyme B, and Fas‐ligand. TCC (0.5 × 10^5^) were cultured with autologous antigen presenting cells (0.1 × 10^5^) in ELISpot plates and cultured for 48 h. Plates were developed using specific secondary antibodies and the secretion of cytokines was visualized as images. C, Chemokine receptor expression analysis. TCC were stained with antibodies for CXCR3-APC, CCR5-PE, CCR1-PE, CCR4-PE, CCR9-APC, CCR8-FITC, CCR6-APC, CLA-FITC, CXCR6-PE, E-Cad-PE, CXCR1, CCR2-APC, and CD69-FITC and receptor expression measured by flow cytometry.

In initial experiments, 3 vancomycin-responsive TCC were generated from vancomycin-treated PBMC (from 2 donors expressing HLA-A*32:01) with no CD4+ or CD8+ enrichment. TCC were stimulated to proliferate with vancomycin in a concentration-dependent manner with different response profiles (Figure 3A). TCC secreted Th1 and Th2 cytokines, IL-10, IL-22 and cytolytic molecules including granzyme B and FasL (Figure 3B). Phenotyping using flow cytometry somewhat surprisingly showed that these TCC were CD4+ and expressed CXCR3 and CCR4 (Figure 3C); thus, in subsequent experiments, magnetic microbeads were used to enrich the number of CD8+ T cells within the vancomycin T-cell lines prior to serial dilution and expansion of TCC.

#### Characterization of Vancomycin-Specific CD8+ TCC Generated From HLA-A*32:01 Positive Drug Healthy Donors

**Figure 4.**
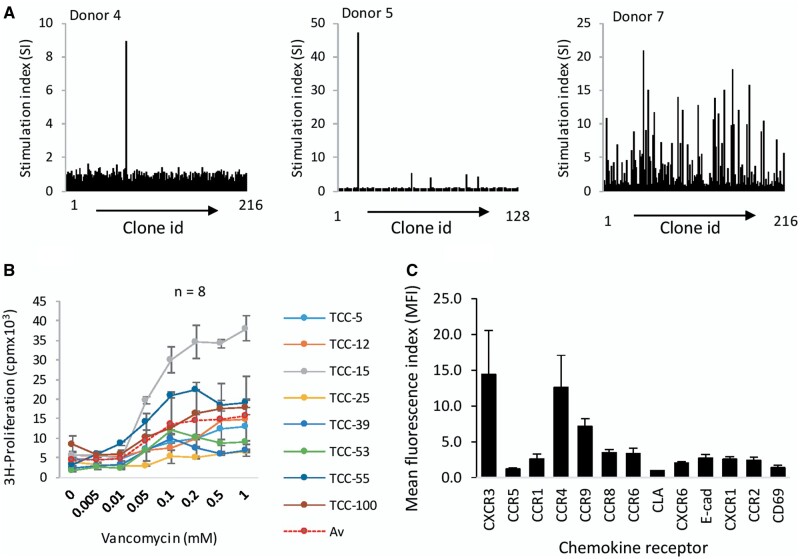
Generation and characterization of vancomycin-responsive CD8+ T-cell clones (TCC) from HLA-A*32:01 positive healthy donors. A, Peripheral blood mononuclear cells (PBMC) from 3 healthy donors were cultured with vancomycin (0.5 mM) for 14 days. TCC were generated from the T-cell lines using serial dilution and repeated mitogen stimulation. Approximately 1 cell per well was seeded into U-bottomed 96-well plates. Growing TCC were selected, expanded and tested for vancomycin specificity. Selected TCC were cultured with autologous antigen presenting cells and vancomycin (0.5 mM) in duplicate cultures for 48 h at 37°C, 5% CO_2_. ^3^H-thymidine (0.5µCi/well) was added for the last 16 h of cell. TCC with stimulation index >2 were expanded for phenotyping and characterization of activation pathway. B, Concentration-dependent proliferation of T cells. TCC (0.5 × 10^5^) from donor 4 (*n* = 1), donor 5 (*n* = 1), and donor 7 (*n* = 6) were cultured with autologous antigen presenting cells (0.1 × 10^5^) and vancomycin in triplicate cultures for 48 h and T-cell activation measured by ^3^H-thymidine incorporation. C, Chemokine receptor expression analysis. TCC were stained with antibodies for CXCR3-APC, CCR5-PE, CCR1-PE, CCR4-PE, CCR9-APC, CCR8-FITC, CCR6-APC, CLA-FITC, CXCR6-PE, E-Cad-PE, CXCR1, CCR2-APC, and CD69-FITC and receptor expression measured by flow cytometry. Data are presented as MFI of mean ± SD values.

Following CD8+ T-cell enrichment, a total of 109 vancomycin-specific TCC were generated from 3 HLA-A*32:01 positive donors (donors 4, 5, and 7) out of 560 TCC tested for vancomycin specificity (Figure 4A). An unprecedented number of vancomycin-responsive TCC were generated from donor 7. Similar to the CD4+ TCC described above, TCC were stimulated to proliferate in a concentration-dependent manner with vancomycin concentrations of 0.05–1 mM activating the TCC (Figure 4B). The strength of the induced proliferative response varied from TCC to TCC; but maximal responses were consistently detected at 0.2 mM and above. TCC expressed high levels of CXCR3 and CCR4, while CCR1, CCR6, CCR8, and CCR9 were expressed lower levels (Figure 4C). Sixty-six of the TCC only expressed CD8+ and these were utilized for the functional assays described below. Supplementary Figure 1 shows CD4+/CD8+ staining on 15 of these TCC. The other TCC expressed CD4+ and CD8+ (dual positive clones) or CD4+ and CD8+ in different populations (indicating that 2 precursor cells were inadvertently added to the initial wells in the serial dilution experiment). These TCC were not studied in detail.

#### Secretion of TC1 and TC2 Cytokines and Cytolytic Molecules From Vancomycin-Responsive CD8+ TCC

**Figure 5.**
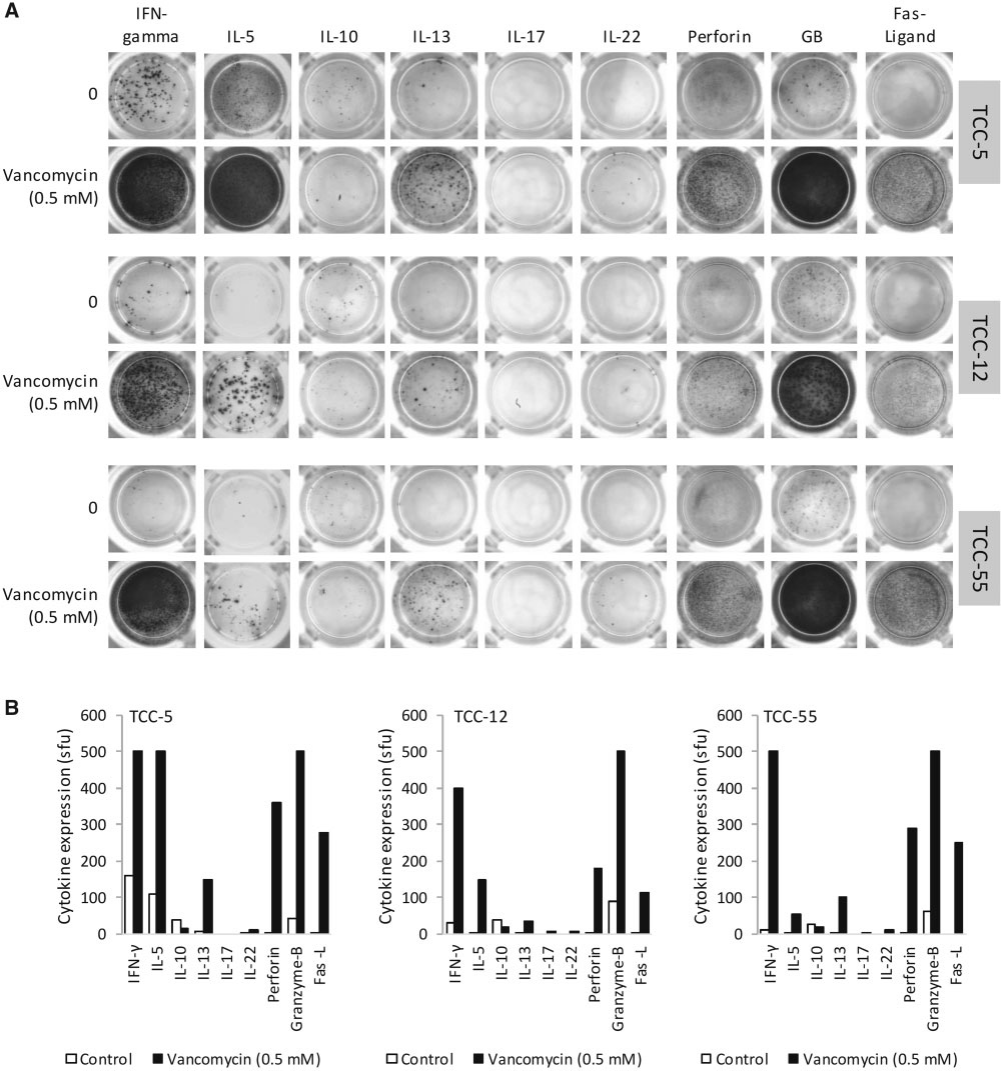
Vancomycin stimulates CD8+ T-cell clones (TCC) to secrete Tc1, Tc2, and cytolytic molecules. A, ELISpot detection of IFN‐γ, IL-5, IL-10, IL‐13, IL‐17, IL‐22, perforin, granzyme B, and Fas‐ligand. Three representative TCC (0.5 × 10^5^) were cultured with autologous antigen presenting cells (0.1 × 10^5^) in ELISpot plates and cultured for 48 h. Plates were developed using specific secondary antibodies and the secretion of cytokines was visualized as images (A) and bar charts showing spot counts (B).

Eight CD8+ TCC were profiled for the secretion of cytokines and cytolytic molecules after vancomycin treatment. TCC were stimulated to secrete a combination of Tc1 (IFN-γ) and Tc2 (IL-5 and IL-13) cytokines alongside cytolytic molecules including perforin, granzyme-B and Fas-ligand. In contrast to the CD4+ TCC, CD8+ TCC did not secrete IL-10, IL-17 or IL-22 (Figs. 5A and 5B). All 8 TCC secreted a similar profile of cytokines. Figure 5 shows 3 out of 8 representative TCC.

#### Activation of CD8+ TCC With Vancomycin and Structurally Related Glycopeptides

**Figure 6.**
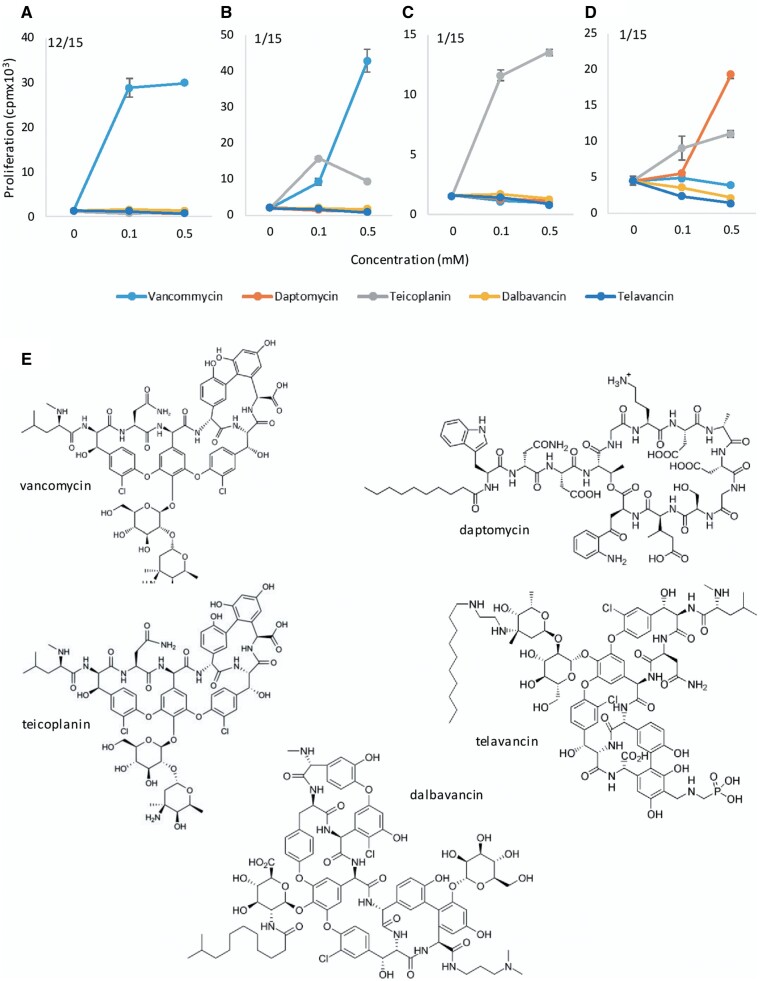
Patterns of activation of vancomycin-responsive CD8+ T-cell clones (TCC). A, Vancomycin-specific TCC; B, Cross-reactive TCC; C, Teicoplanin-responsive TCC; and D. Daptomycin-responsive TCC. TCC were cultured with autologous antigen presenting cells in the presence of vancomycin, teicoplanin, daptomycin, dalbavancin, and telavancin (0.01–0.5 mM) for 48 h followed by ^3^H-thymidine incorporation to determine drug-specific T-cell activation. A total of 15 TCC were profiled. E, Structure of the glycopeptides.

To investigate cross-reactivity of vancomycin-responsive TCC with other glycopeptides, 15 TCC were cultured with either daptomycin, teicoplanin, dalbavancin, or telavancin. The majority of the TCC (80%) were specific to vancomycin (Figure 6A). One TCC displayed additional reactivity with teicoplanin (Figure 6B). Interestingly, 2 unexpected patterns of reactivity were observed with 2 TCC previously weakly responsive to vancomycin: (1) 1 TCC showed a strong proliferative response to teicoplanin (Figure 6C) and (2) 1 TCC proliferated in responsive to daptomycin and teicoplanin (Figure 6D). None of the vancomycin-responsive TCC was activated with either dalbavancin or telavancin.

#### MHC Class I-Restricted Activation of CD8+ Vancomycin-Specific TCC

**Figure 7.**
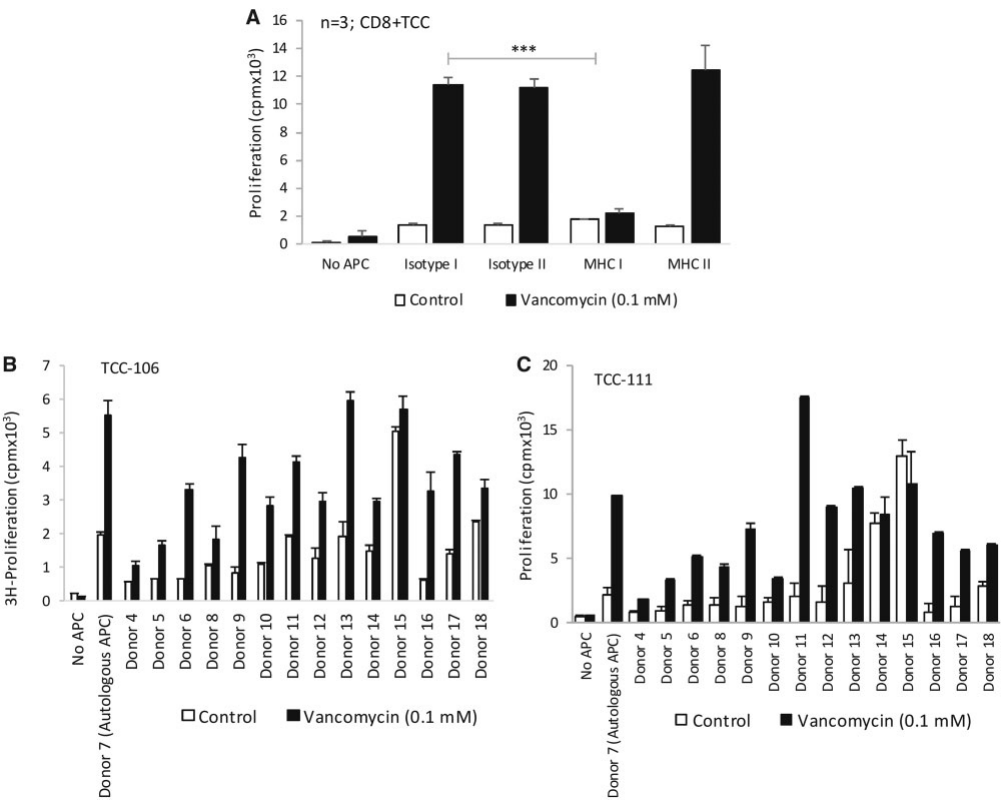
MHC class I-restricted activation of CD8+ vancomycin-specific T-cell clones (TCC). A, Vancomycin-specific TCC were cultured with autologous antigen presenting cells and vancomycin (0.1 mM) in the presence of isotype I (MHC class I), isotype II (MHC class II), MHC classes I or II blocking antibodies for 48 h. B and C, TCC were cultured with either autologous or heterologous antigen presenting cells and vancomycin for 48 h and T-cell proliferation determined by 3H-thymidine incorporation. HLA genotype of the donor antigen presenting cells is shown in Table 1.

CD8+ TCC displaying proliferation exclusively in the presence of EBV-transformed B cells (ie, TCC with no self-presentation) were used to explore the importance of MHC classes I and II molecules in T-cell activation. Activation of TCC with vancomycin and autologous EBV-transformed B cells was inhibited with an anti-human MHC class I blocking antibody. In contrast, the proliferative response in the presence of the antihuman MHC class II blocking antibody was the same as observed with the isotype control (Figure 7A).

A panel of EBV-transformed B cells generated from human donors expressing HLA-A*32:01 or dissimilar HLA-A alleles were used to investigate the importance of HLA-A*32:01 in the T-cell proliferative response. Table 1 shows the HLA classes I and II molecules expressed by the different donors. TCC were stimulated to proliferate in the presence of either the autologous (donor 7) or heterologous EBV-transformed B cells (*n* = 8; donors 4–13) expressing HLA-A*32:01 (Figs. 7B and 7C). TCC also displayed proliferative responses in the presence of vancomycin and EBV-transformed B cells from donors 16 ([A*01:01/A*02:01] and 17 [A*11:01/A*30:01]). Alloreactivity was observed with EBV-transformed B cells from donors 14 and 15, so it was difficult to determine the extent of vancomycin-induced proliferation. Results from 2 representative vancomycin-responsive TCC are shown in Figures 7B and 7C.

#### Direct, Processing-Independent Activation of Vancomycin-Responsive CD4+ and CD8+ TCC

**Figure 8.**
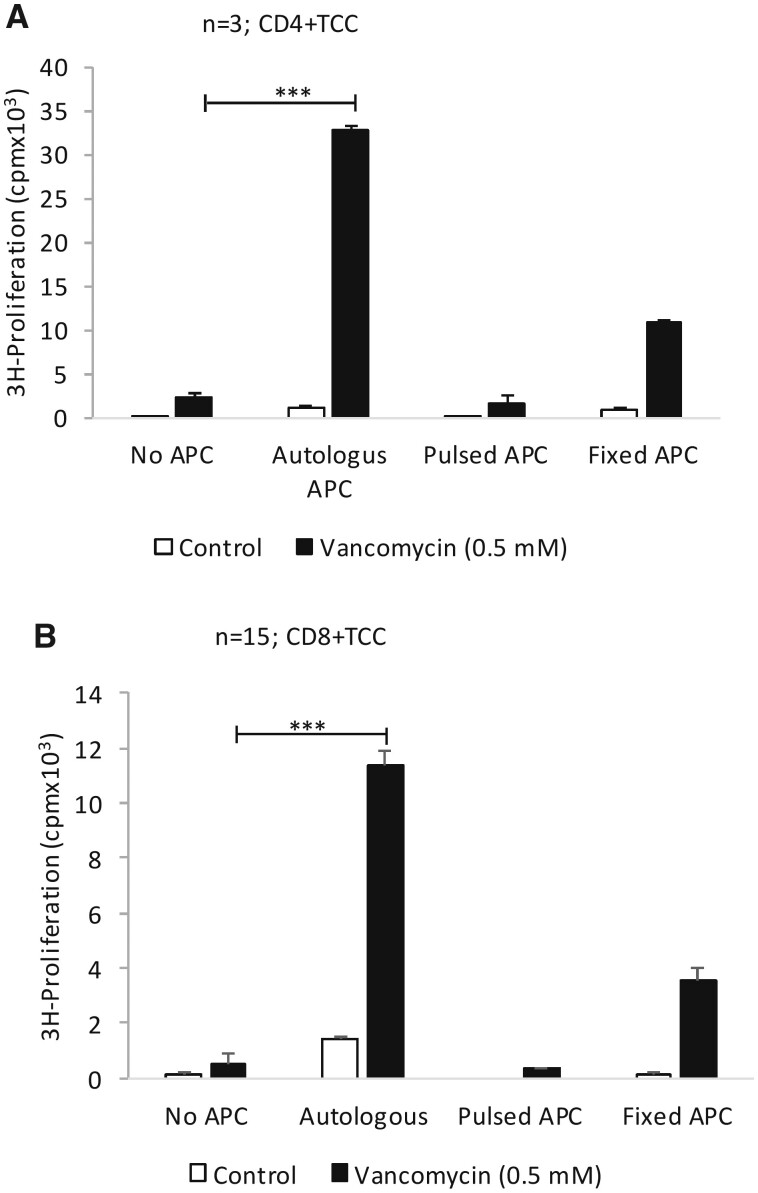
Direct processing-independent activation of CD4+ and CD8+ T-cell clones (TCC) with vancomycin. A, CD4+ and (B) CD8+ TCC were cultured with irradiated or glutheraldehyde-fixed antigen presenting cells and vancomycin for 48 h and drug-specific T-cell proliferation determined by ^3^H-thymidine incorporation. TCC were also cultured antigen presenting cells that had been pulsed with vancomycin for 16 h. The vancomycin pulsed antigen presenting cells were washed (to remove nonbound vancomycin), irradiated and cultured with TCC for 48 h in the absence of soluble drug. Student’s *t* test was performed to determine statistical significance of T-cell proliferation (*p ≤ .05; **p ≤ .005; ***p < .001).

Proliferation of CD4+ and CD8+ TCC was not detected when they were cultured with EBV-transformed B cells pulsed with vancomycin for 16 h (Figure 8). However, glutaraldehyde fixed B cells that no longer process proteins into peptide fragments, stimulated CD4+ and CD8+ TCC to proliferate when soluble vancomycin was present. TCC exposed to soluble vancomycin and irradiated B cells were used as a positive control (Figure 8).

## DISCUSSION

Drugs designed as therapeutics are sometimes associated immune-mediated adverse events that develop into tissue and organ damage in a small percentage of patients. Multiple retrospective GWAS have identified expression of single or a small number of HLA alleles as a critical but not absolute susceptibility factor for certain forms of reaction. The recent identification of HLA-A*32:01 allele expression as a susceptibility factor for vancomycin-induced DRESS strongly implicates the adaptive immune system in the disease pathogenesis and suggests that the drug may interact either directly or indirectly with the protein encoded by HLA-A*32:01 to activate drug-specific T cells (Konvinse *et al.*, 2019). The relatively high frequency of HLA-A*32:01 among individuals of European ancestry underscores the scale of potential problem with vancomycin administration. PBMC isolated from patients 12/13 patients with vancomycin-induced DRESS were shown to secrete IFN-γ when cultured *in vitro* with the drug. Furthermore, molecular docking of vancomycin with HLA-A*32:01 suggests that the drug binds to the HLA peptide binding cleft with higher affinity in the absence of a peptide (Konvinse *et al.*, 2019), which raises the possibility that the drug may act as a peptidomimetic to activate CD8+ T cells.

This study utilizes our established HLA-typed biobank of healthy donors (Alfirevic *et al.*, 2012) to investigate whether expression of HLA-A*32:01 is critical for the priming naïve of T cells with vancomycin. Vancomycin-responsive TCC were then generated from the HLA-A*32:01+ donors to (1) determine whether vancomycin exclusively activates CD8+ T cells, (2) define cellular phenotype and function of vancomycin-responsive T cells and (3) explore pathways of drug presentation and cross-reactivity with structurally related drugs. The priming assay employed involves culturing naïve T cells with autologous dendritic cells and vancomycin in multiple wells of a 96-well culture plate. After 14 days in culture half of the primed T cells were restimulated with vancomycin, whereas the other wells served as negative controls. Vancomycin primed naïve T cells from all 3 donors (donors 4–6) that expressed HLA-A*32:01, with over half of the drug-treated wells displaying proliferative responses. Significantly, priming of naïve T cells with vancomycin was also observed with cells from 2 out of 3 donors expressing HLA alleles other than HLA-A*32:01(donors expressing HLA-A*02:30/A*31:01 [donor 2] and A*24:02/A*25:01 [donor 3]). To explore the activation of PBMC from drug naïve donors in greater detail, PBMC from 10 HLA-A*32:01 positive to 10 HLA-A*32:01 negative donors were cultured with vancomycin for a period of 4 weeks. The drug-treated cultures were restimulated weekly with a cocktail consisting of vancomycin, irradiated autologous PBMC (as antigen presenting cells) and IL-2, and drug-specific proliferative responses were measured at the end of the culture period. Wells containing vancomycin-responsive T cells were detectable in 7/10 HLA-A*32:01 positive donors and only 1 HLA-A*32:01 negative donor. These data indicate that although vancomycin may have a binding preference for HLA-A*32:01 or peptides embedded within the HLA antigen binding groove, it interacts with additional HLA molecules or HLA peptide complexes. HLA peptide elusion studies, X-ray crystallography as well as *in silico* and *in vitro* analysis is required to further understand the interaction between vancomycin, HLA-A*32:01, HLA-binding peptides, and specific T-cell receptors.

The GWAS study linking vancomycin-induced DRESS to HLA-A*32:01 implicates CD8+ cells in disease pathogenesis as HLA class I molecules preferentially present peptide and drug antigens to CD8+ T cells. For this reason we generated vancomycin-responsive TCC from vancomycin-enriched T-cell lines and characterized their CD phenotype. In initial experiments, 3 vancomycin-response TCC were generated from 2 donors. All 3 TCC were stimulated to proliferate and secrete Th1 and Th2 cytokines, IL-17 and IL-22 following exposure to vancomycin and autologous antigen presenting cells. Unexpectedly, these TCC expressed the CD4 surface receptor. Nakkam *et al.* (2021) recently used molecular docking to show that glycopeptide antibiotics including vancomycin, teicoplanin, and telavancin, which displayed a degree of T-cell cross-reactivity in a DRESS patient bind to HLA class II molecules such as HLA-DQA1*01:01, HLA-DQB105:03. This led the authors to propose that cross-reactivity between the drugs may involve HLA class II presentation to CD8+ T cells or alternatively activation of CD4+ T cells. Our T-cell cloning data unequivocally demonstrates that vancomycin activates CD4+ T cells in donors carrying HLA-A*32:01.

To explore whether vancomycin-responsive CD8+ TCC were detectable in HLA-A*32:01+ donors, the cloning protocol was modified to select CD8+ T cells prior to establishing serial dilution plates. Vancomycin-responsive CD8+ TCC were detected from all 3 donors studied. TCC that proliferated in the presence of vancomycin were detected in considerably different numbers from each donor. The reason for this difference is unclear as all the donors expressed HLA-A*32:01 and TCC were generated from cell lines exposed to the same concentration of vancomycin. It is plausible that differences may relate to differential expression and activity of coinhibitory receptors or a diverse expression of vancomycin-responsive T-cell receptor sequences. Both of these areas of research warrant further investigation.

To investigate vancomycin-specific TCC in greater detail, TCC were first cultured with graded concentration of vancomycin. Proliferative responses were detected with concentration of vancomycin as low as 0.05 mM, with maximal responses plateauing at 0.2 mM. These concentrations are only slightly above the plasma concentrations of 0.01–0.02 mM needed to exert an on-target pharmacological effect patients (Tunkel *et al.*, 2004; Wysocki *et al.*, 2001). TCC expressed high levels of the chemokine receptors CXCR3 and CCR4. The interaction of CXCR3 with its ligands (CXCL9 and CXCL10) results in the trafficking of activated effector CD8+ T cells to target sites including skin where they are involved in Th1 cytokine-mediated inflammation (Groom and Luster, 2011; Maurice *et al.*, 2019). CCR4+ CD8+ T-cell subsets are relatively rare; they migrate in the presence of CCL17 and CCL22 to inflammatory cutaneous sites prior to activation (Kondo and Takiguchi, 2009). Similar to the vancomycin-responsive CD4+ T cells, the CD8+ TCC secreted IFN-γ, IL-5, IL-13 and cytolytic molecules (perforin, granzyme B and Fas ligand), which may play a role in the pathogenesis of DRESS.

The molecular weight of vancomycin (1449.2) is significantly larger than most small molecule drugs (molecular weight often <500, Zhao *et al.*, 2012) and more closely resembles the weight of an HLA class I binding peptide. As discussed earlier, it is possible that the binding affinity of vancomycin towards HLA-A*32:01 may result in the displacement of HLA bound peptides displayed by the HLA molecule on the surface of antigen presenting cells. To study whether activation of TCC with vancomycin was dependent on antigen processing within antigen presenting cells and/or the formation of covalently bound vancomycin protein adducts, the TCC were cultured with (1) drug, antigen presenting cells and HLA blocking antibodies (2) drug and glutaraldehyde-fixed antigen presenting cells (fixation blocks processing but not presentation of preprocessed antigens, Zanni *et al.*, 1998), and (3) antigen presenting cells pulsed with vancomycin for 16 h (washing removes weakly associated drugs from the HLA peptide binding cleft (Schnyder *et al.*, 2000)). All vancomycin-responsive CD8+ TCC were activated through a reversible pharmacological interaction of the drug with HLA class I molecules via a pathway that did not require antigen processing within the antigen presenting cells. HLA-mismatching experiments showed that TCC were activated in the presence of multiple heterologous antigen presenting cells expressing HLA-A*32:01. However, proliferative responses were also observed when the TCC were cultured with vancomycin and antigen presenting cells from certain donors expressing other HLA-A alleles. These data with vancomycin differ from the results of similar experiments studying flucloxacillin- and abacavir-specific HLA-B*57:01-restricted T-cell responses, where T-cell activation was only detected with antigen presenting cells expressing HLA-B*57:01 (Chessman *et al.*, 2008; Monshi *et al.*, 2013), and indicate that vancomycin forms complexes with several HLA molecules and/or HLA bound peptides. However, further mechanistic and structural studies are needed using cell lines stably transfected with HLA-A*32:01 to shed light on the detailed molecular interaction of vancomycin with HLA, HLA binding peptides and specific T-cell receptors.

Tiecoplanin (molecular weight [MW] = 1879.7), daptomycin (MW = 1619.7), dalbavancin (MW =1816.7), and telavancin (MW = 1755.6) are all glycopeptide antibiotics used to manage patients with antibiotic resistant Gram-positive microbes. Hence, we investigated whether vancomycin-responsive CD8+ TCC were activated by these drugs. Although majority of the TCC were vancomycin-specific, 1 TCC was cross reactive with teicoplanin and intriguingly, 2 TCC weakly responsive towards vancomycin were stimulated with either teicoplanin or both teicoplanin and daptomycin. These data suggest that our experiments identified a small number of TCC that display preferential activation with alternative glycopeptide antibiotic structures. The implications of this are unclear and additional mechanistic investigations are needed to define the drug interaction at the immunological synapse between HLA-A*32:01 and specific T-cell receptors. Teicoplanin has similar bacterial efficacy to vancomycin and has been used as an alternative in cases of vancomycin intolerance; however, its use in this patient group is associated with a high degree of adverse events (Hsiao *et al.*, 2012), suggesting possible cross-reactivity between the 2 drugs. Our data show that this clinical cross-reactivity may relate to the activation of a small number of vancomycin-response CD8+ TCC with teicoplanin. In on-going experiments we are using the T-cell priming assay described herein to explore the intrinsic immunogenicity of the different glycopeptide antibiotics and to determine whether priming to each structure is more efficient in donors expressing HLA-A*32:01.

To conclude, this study provides evidence that vancomycin primes naïve T cells from healthy donors expressing HLA-A*32:01, an HLA allele strongly associated with vancomycin-induced DRESS, through a direct pharmacological binding interaction. Vancomycin stimulates CD4+ and CD8+ T cells to proliferate and secrete an abundance of cytokines and cytolytic molecules. Cross-reactivity of the vancomycin-responsive T cells with teicoplanin provides an explanation for the teicoplanin reactions observed in vancomycin hypersensitive patients. The absence of cross-reactivity with other drugs suggests that these may be safer alternatives in vancomycin hypersensitive patients.

## SUPPLEMENTARY DATA

Supplementary data are available at *Toxicological Sciences* online.

## Supplementary Material

kfab084_Supplementary_Data
