# Supplementary material for: Deciphering Adverse Drug Reactions: In Vitro Priming and Characterization of Vancomycin-Specific T Cells From Healthy Donors Expressing HLA-A*32:01
Source: Toxicol Sci. 2021 Jun 27;183(1):139–53. doi: 10.1093/toxsci/kfab084 (PMC8404995; doi:10.1093/toxsci/kfab084)
Supplement: kfab084_Supplementary_Data [file kfab084_supplementary_data.zip › 20200525 Online supplementary material-1 clean.docx]

Treatment Delay and Reperfusion Management of Acute ST-segment Elevation Myocardial Infarction in Tertiary PCI Hospitals: analysis of the China STEMI Care Project Phase 1 (CSCAP-1)

Authors: Yan Zhang,^1^ MD, Ye Tian,^2^ MD, Pingshuan Dong,^3^ MD, Yawei Xu,^4^ MD, Bo Yu,^5^ MD, Hui Li,^6^ MD, Jifu Li,^7^ MD, Junbo Ge,^8^ MD, Yuhong Sun,^9^ MD, Jianan Wang,^10^ MD, Lefeng Wang,^11^ MD, Jiyan Chen,^12^ MD, Hongbin Yan,^13^ MD, Yundai Chen,^14^ MD, Yaling Han,^15^ MD, Yong Huo,^1^ MD, on behalf of the CSCAP-1 Investigators

**Contents:**

Table S1 Characteristics of the patients with STEMI stratified by in-hospital survival status

Table S2 Characteristics of the patients with STEMI stratified by cardiac function at presentation

Figure S1 Geographical distribution of hospitals participating in CSCAP-1

**Table S1 Characteristics of the patients with STEMI stratified by in-hospital survival status**

|  | Discharge | | Death | | p | |
| --- | --- | --- | --- | --- | --- | --- |
| n | 4090 | | 101 | |  | |
| Age, y | 60.6±12.4 | | 71.0±9.8 | | <0.001 | |
| Female, % | 831 (20.3) | | 47 (46.5) | | <0.001 | |
| **Complication** |  | |  | |  | |
| Previous myocardial infarction, % | 194 (4.7) | | 6 (5.9) | | 0.577 | |
| Hypertension, % | 2053 (50.2) | | 47 (46.5) | | 0.467 | |
| Diabetes, % | 932 (22.8) | | 32 (31.7) | | 0.036 | |
| **Presenting condition** |  | |  | |  | |
| Systolic blood pressure, mmHg | 127.3±24.0 | | 113.6±24.6 | | <0.001 | |
| Diastolic blood pressure, mmHg | 78.2±14.9 | | 71.0±16.8 | | <0.001 | |
| Heart rate, beats per minute | 76.2±16.2 | | 85.3±20.6 | | <0.001 | |
| **Cardiac function, %** |  | |  | | <0.001 | |
| Level I | | 3152 (77.1) | | 47 (46.5) | | |
| Level II | | 719 (17.6) | | 18 (17.8) | | |
| Level III | | 91 (2.2) | | 15 (14.9) | | |
| Level IV | | 128 (3.1) | | 21 (20.8) | | |
| **Methods of arriving in hospitals** |  | |  | | 0.306 | |
| Calling EMS, % | | 1077 (26.3) | | 33 (32.7) | |  |
| Other hospitals, % | | 1003 (24.5) | | 25 (24.8) | |  |
| By themselves, % | | 2010 (49.1) | | 43 (42.6) | |  |
| **Reperfusion strategy (%)** |  | |  | | <0.001 | |
| Non reperfusion | | 414 (10.1) | | 23 (22.8) | | |
| Primary PCI | | 3444 (84.2) | | 73 (72.3) | | |
| Thrombolysis | | 232 (5.7) | | 5 (5.0) | | |
| Length of stay, days | 9.0 [7.0, 12.0] | | 3.0 [1.0, 7.0] | | <0.001 | |

EMS, emergency medical services; PCI, percutaneous coronary intervention

**Table S2 Characteristics of the patients with STEMI stratified by cardiac function at presentation**

|  | Non cardiogenic shock | | | | | Cardiogenic shock | p | | |
| --- | --- | --- | --- | --- | --- | --- | --- | --- | --- |
| n | 4042 | | | | | 149 |  | | |
| Age, y | 60.7±12.4 | | | | | 66.2±12.7 | <0.001 | | |
| Female, % | 836 (20.7) | | | | | 42 (28.2) | 0.027 | | |
| **Complication** |  | | | | |  |  | | |
| Previous myocardial infarction, % | 191 (4.7) | | | | | 9 (6.0) | 0.460 | | |
| Hypertension, % | 2034 (50.3) | | | | | 66 (44.3) | 0.149 | | |
| Diabetes, % | 926 (22.9) | | | | | 38 (25.5) | 0.460 | | |
| **Presenting condition** |  | | | | |  |  | | |
| Systolic blood pressure, mmHg | 127.9±23.5 | | | | | 101.9±27.1 | <0.001 | | |
| Diastolic blood pressure, mmHg | 78.5±14.7 | | | | | 64.7±17.7 | <0.001 | | |
| Heart rate, beats per minute | 76.2±15.7 | | | | | 82.4±29.2 | <0.001 | | |
| **Methods of arriving in hospitals** |  | | | | |  | 0.057 | | |
| Calling EMS, % | 1058 (26.2) | | | | | 52 (34.9) |  | | |
| Other hospitals, % | 994 (24.6) | | | | | 34 (22.8) |  | | |
| By themselves, % | 1990 (49.2) | | | | | 63 (42.3) |  | | |
| **Reperfusion strategy (%)** |  | | |  | | | | 0.221 |  |
| Non reperfusion | | 420 (10.4) | | | 17 (11.4) | | |  |  |
| Primary PCI | | 3398 (84.1) | | | 119 (79.9) | | |  |  |
| Thrombolysis | | 224 (5.5) | | | 13 (8.7) | | |  |  |
| **Outcomes** | | |  |  | | | |  |  |
| In hospital mortality, % | 80 (2.0) | | | | 21 (14.1) | | | <0.001 | |
| Length of stay, days | 9.0 [7.0,12.0] | | | | 11.0 [7.0,17.0] | | | <0.001 | |

EMS, emergency medical services; PCI, percutaneous coronary intervention

**Figure S1** **Geographical distribution of hospitals participating in CSCAP-1**
